# Metaphors in search of a target: the curious case of epigenetics

**DOI:** 10.1080/14636778.2015.1034849

**Published:** 2015-05-12

**Authors:** Aleksandra Stelmach, Brigitte Nerlich

**Affiliations:** ^a^ School of Sociology and Social Policy, Institute for Science and Society, University of Nottingham, Nottingham, UK

**Keywords:** epigenetics, metaphors, media

## Abstract

Carrying out research in genetics and genomics and communicating about them would not be possible without metaphors such as “information,” “code,” “letter” or “book.” Genetic and genomic metaphors have remained relatively stable for a long time but are now beginning to shift in the context of synthetic biology and epigenetics. This article charts the emergence of metaphors in the context of epigenetics, first through collecting some examples of metaphors in scientific and popular writing and second through a systematic analysis of metaphors used in two UK broadsheets. Findings show that while source domains for metaphors can be identified, such as our knowledge of electrical switches or of bookmarks, it is difficult to pinpoint target domains for such metaphors. This may be indicative both of struggles over what epigenetics means for scientists (natural and social) and of difficulties associated with talking about this, as yet, young field in the popular press.

## Introduction

For over three decades linguists and social scientists have observed and described how advances in genetics and genomics have been made public in the media, from cloning and the creation of Dolly the sheep in 1997 to synthetic biology and the creation of the first synthetic cell in 2010 and beyond (Nelkin and Lindee 1995; Condit 1999; Nerlich and Hellsten 2004; Nerlich and Hellsten 2009; Boudry and Pigliucci 2013; Rossi 2013; De Lorenzo 2014). They have sifted through increasing flotsam and jetsam of metaphors washed up on the beach of knowledge by each new wave of discovery (alongside each new wave of hype). In this article, we examine how the emergence of epigenetics – a field of genetics that studies phenomena “beyond” (epi-) genes and genomes – contributes to changing the science of genes and humans but also contributes to changing the metaphorical landscape created by doing this science and talking about it in public. We ask: How is epigenetics framed metaphorically in the popular press and what are the scientific and political implications of this framing?

Epigenetics is a new field of science (Landecker and Panofsky 2013) and a new scientific movement. As Haig has pointed out:

Epigenetics has clearly provided a banner under which a new scientific movement has advanced. At the heart of this movement is research on the role of chromatin modification in the control of transcription. But the movement is a broad tent that unites studies of the effects of environmental toxins on gene expression, of the fetal origins of adult disease and of how early rearing affects adult behaviour. The indefinite definition of epigenetics (together with the connotation of being ‘above’ or ‘beyond’ genetics) has meant that scientists from divergent disciplines, studying only loosely related phenomena, could all feel they were engaged in epigenetic research near the cutting edge of modern biology. (Haig 2012, 15)

Epigenetics is also becoming an object of study for social scientists and an object of speculation for policy-makers, including speculations about new ways of envisioning links between nature and nurture, about eliminating poverty, abuse, violence, mental illness, cancer and much more (see Newitz 2013; Pickersgill et al. 2013; Juengst et al. 2014; Meloni and Testa 2014; Richardson et al. 2014).

Despite becoming fashionable only recently, epigenetics has a long history which is intertwined with the history of genetics and genomics, developmental biology, zoology and embryology. After first being proposed by Conrad Hal Waddington in the 1940s and David Ledbetter Nanney in the 1950s, the concept of epigenetics has begun to change (Haig 2004; Riddihough and Zahn 2010). Since the 1990s, a new “molecular epigenetics” has emerged as the study of changes in the genome that do not involve changes in the DNA sequence, alongside multiple new branches and specialisms, such as environmental epigenetics, behavioral epigenetics and cancer epigenetics, to name just a few. Overall, epigenetics is opening up new ways of investigating the complex interactions between social phenomena, human behavior and human biology or, as some might say, between nature and nurture.

In the process, the meaning of epigenetics has become, as some claim, surrounded by “a muddle” (Keller 2010, 5). Most definitions of epigenetics mention heritable changes within a cell that do not result from changes in DNA sequence itself but are triggered by environmental factors (see Berger et al. 2009). However, as Adrian Bird from the University of Edinburgh recently told *New Scientist*: the term actually encompasses “a vast array of molecular mechanisms that affect the activity of genes” (Bird 2013a, i). In this article, we keep both this definition and Keller's “muddle” in mind when studying how epigenetics is discussed in the press. Can we find reflections of a muddle there or does one view of epigenetics predominate?

Our interest in this article is in how the linguistic landscape of this field of science is configured in the press through the use of metaphors and what this may mean for public understanding, for politics and social policy and, of course, also for the sociological study of science. In the following we first provide a brief overview of older genetic and genomic metaphors and then go on to explore emerging metaphors related to epigenetics.

## Metaphors in genetics and genomics

Genetics and genomics have, in the past, been dominated by several interrelated clusters of metaphors, which have framed the ways genes are studied as well as the ways issues around genetics and genomics are communicated. Popular genetics and genomics discourses were built around a small number of what one can call “grand” metaphors relating to master narratives about what makes us human (Lyotard 1979), such as the book of life, the blueprint of life, the glorious map, as extolled in a speech by Bill Clinton when the first draft of the human genome was revealed in the year 2000 (Nerlich, Dingwall, and Clarke 2002) and, of course, the code and computer program of life. The hope expressed in these grand narratives was that they would reveal what it means to be human. We ask whether epigenetics is framed by similar grand metaphors and grand narratives and, if not, what this means for this field of study and the claims built up around it.

Alongside these grand metaphors, various grand claims were made in the 1990s with respect to genetics and genomics, especially in the popular and social science literature, namely that genes determine our destiny, that genes have power and that we live in an age of genetic determinism (Nelkin and Lindee 1995). This has led to people blaming their genes, for example, for obesity, rather than the environment or individual actions. Alongside these claims, promises were made about the advent of personalized medicines. Francis Collins, for example, the American lead on the human genome project, published a book in 2010 entitled *The Language of Life: DNA and the Revolution in Personalized Medicine* (Collins 2010). In popular genetics and genomics, the direction of claims being made through the use of metaphors was from the inside to the outside. The story went that if you change or enhance your genes, you become a better or healthier person. We will see how this directionality changes with the advent of epigenetics.

Since the decipherment of the human genome, which unsettled rather than settled some popular tenets (and metaphors) of genetics, there have been proposals to use different sets of metaphors (Avise 2001) in order to highlight the many non-deterministic aspects of genetics and genomics. In fact, it is quite clear to genetic and genomic scientists that most of genetics and genomics are probabilistic and not deterministic in nature (see Zwart 2009). In his seminal book *The Music of Life*, the physiologist and one of the founding fathers of systems biology Noble (2006) argued, and still argues today in the context of epigenetics, that a gene-centric view in biology should be replaced by an integrative and systems-based one. He tried to highlight this shift of perspective by replacing the book and code metaphors with a music-based metaphor (the music of life), which has become even more popular within discourses about epigenetics, as we shall see.

Genetic and genomic metaphors began to change around the same time as the human genome was finally deciphered in full, in 2003. This was also the year that the Human Epigenome Project was launched (Bradbury 2003). There was a shift in language, from establishing maps, deciphering codes, reading books or blueprints, to writing books or codes, and building, designing or engineering new blueprints for life. Alongside this new more dynamic language, a new realism was emerging. At the beginning of the millennium, hopes were high that the secret of the human genome was finally revealed and politicians thought scientists would be able “to speak the language of God” (Nerlich, Dingwall, and Clarke 2002). This was immediately followed by the realization that much less was known about the genome than previously thought, especially about how genes function, how they are regulated and so on – a gap in knowledge that epigenetics is trying to fill. Hopes of reading or even writing “the book of life” are tempered by a realization that scientists still do not know enough about the genome's “dark matter.” This recent metaphor encapsulates a shift in scientific interest away from “genes” to “junk DNA” which makes up large parts of the genome and also to epigenetic phenomena (Nerlich 2014; Carey forthcoming).

**Figure 1.**
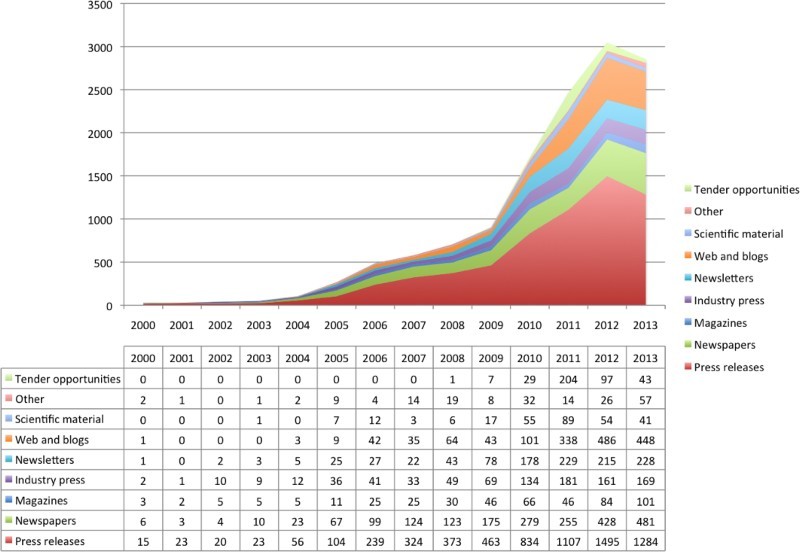
“Epigenetics” in All English Language News (Nexis^®^).

As Figure 1 shows, the media gradually started to increase their attention to epigenetics after 2003 and the pace of research and reporting picked up after 2006 and 2008, with press releases driving these developments, together with trade and industry press and letter web-based publications. In 2008, the National Institutes of Health pledged $190 million to map the epigenetic “marks” on the human genome (*NIH News* 2008). While scientific publications have increased steadily over time with an eight-fold increase from just over 1000 papers in 1992 to more than 8500 in 2011(Cherfas 2013), the mainstream media still have to catch up with these developments.

**Figure 2.**
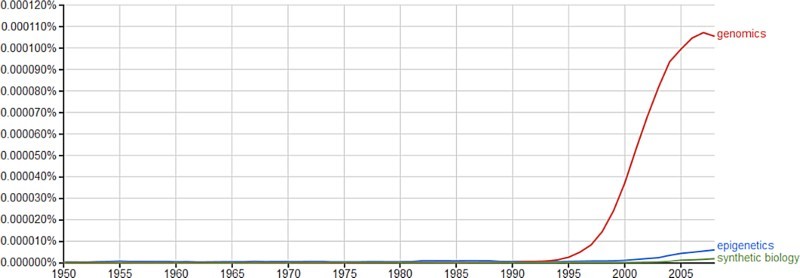
Genomics, synthetic biology and epigenetics on Google Ngram viewer (Google books).

What some call the “epigenetic revolution” (Carey 2012) has to be kept in perspective though, as Figure 2, derived from Google Ngram viewer (https://books.google.com/ngrams), shows. Genomics is still the main topic of research since around 1995 (and the term post-genomics, which is so often used in conjunction with epigenetics in social science literature, does not really get a look-in).

We shall now examine how epigenetics extended the genetic and genomic repertoire of metaphors. We do this in two steps. First we provide a rather impressionistic and non-systematic account of some interesting metaphors we have found while engaging with the issue of epigenetics and metaphors. The sample of metaphors found quite randomly in scientific articles, blogs etc. therefore only provides a glimpse of what is out there and future research will have to be much more systematic in this respect. In a second step, we engage in the systematic analysis of a small sample of newspaper articles. Again this is only a small sample and future research will have to go beyond what we could do here in this short article.

## Emerging metaphors for epigenetic phenomena

It is almost impossible to survey the rapidly increasing scientific and media output around epigenetics thoroughly enough to extract a definitive list of old, changed or new metaphors (see Robison 2014). In the following we shall first provide some initial impressions and a rather haphazard selection of metaphors that have washed up on our epigenetic beach so to speak. We shall deal with them like naturalists in the past did with the butterflies and fossils they collected, that is, use them as indications of what might be going on in our shifting metaphorical landscape. After this, we shall home in more systematically and methodically on metaphors used in a small sample of UK news articles.

Four metaphors were central to making genetics and genomics public: the book, code/program, map and blueprint metaphors and variations on these themes (see Nerlich and Hellsten 2004). From a first glance at some manifestations of the new epigenetics discourse, it seems that these metaphors are still used, but that they are also creatively adapted to the needs of this new field. However, as we shall see in our small media analysis, alongside these grand metaphorical narratives, or rather, underpinning them, there are a host of “smaller” metaphors which make epigenetics discourses special.

Book metaphors are still in use, but the focus has shifted in accordance with advances in genomics and synthetic biology, from mere reading to more dynamic writing (Hellsten and Nerlich 2011). To give just a few examples: “DNA, like a **book**, is organised into modules. ( … ) The epigenetic **machinery** is in charge of determining the accessibility of the **pages** to the **readers** of DNA” (Calvanese, Lara, and Fraga 2012).^1^ More creatively: “Epigenetics is the **coffee stain on the page** that gets copied when you photocopy the book, and when someone photocopies your copy” (Boyle 2011). Or: “Genetics and Epigenetics – **Nature's Pen-and-Pencil** **Set**” (Gosden and Feinberg 2007). More creatively still:

scientists now know that genes are not the only authors of inheritance. There are **ghostwriters**, too. At first glance, these scribes seem quite ordinary – methyl, acetyl, and phosphoryl groups, clinging to proteins associated with DNA, or sometimes even to DNA itself, looking like **freeloaders** at best. (Rogers 2012)

In a sense, the book of life really comes to life here!

The blueprint metaphor too is not dead yet. An article advertised on the cover of *New Scientist* was entitled “Epigenetics: The other **blueprint** of life” (New Scientist, 5 January 2013). One European project examining the epigenetics of blood cells and a follow-up to the Human Genome Project has chosen “**BLUEPRINT**” as its title (Feilden 2011).

As genes and environment are now more closely linked, there are newer metaphors that talk about the epigenome as a memory bank, storing traces of past experiences which may influence future generations. We shall look at these metaphors more closely in the next section.

Music metaphors seem to be more prominent in descriptions of epigenetics than in older genetic and genomic discourses, and one can find many references to keyboards, juke boxes and pianos. This is in a way quite understandable, as epigenetics seems to demonstrate the plasticity, flexibility and variability of “the book of life.” To give only two examples: “In biological terms the **pianist** corresponds to the epigenetic processes that ‘**play**' the otherwise static linear information represented in DNA” (Klinghoffer 2012).

The music metaphor is perhaps the most promissory metaphorical framing of epigenetics, opening up a conceptual space for arguing that we can now change our genetic fate, to play a different tune if you like (see Bateson 2001). It is linked to the issue of programming and plasticity. We can, so some say, shape not only who “we” want to be but how we want future generations to be and how healthy or unhealthy they can be. Some social scientists even speculate about alleviating the negative health impacts of inequalities or class differences (Hedlund 2012; Loi, del Savio, and Stupka 2013). Here politics and social engineering creep into the epigenetic landscape and with them perhaps a new form of “somatic determinism” as Margaret Lock warns us (Lock 2013). Or as some say: “Epigenetics is the new ‘gene for’” (Buchanan 2013). There is a danger then of both hype and a return to out-dated framing in terms of determinism.

Alongside this promissory framing, there are also some negative images surrounding epigenetics which find expression in metaphors such as “the **ghost** in our genes,” “Grandma's **curse**,” “womb **doom**,**”** “**sins** of the father,” “**poison** that keeps poisoning through the generations” or “a **time bomb** in your genes” (see Inglis-Arkell 2011; *The Economist* 2012). Both the positive and negative metaphorical framing need of course more detailed investigation in the future.

We shall now home in on a small corpus of newspaper articles extracted from *The Guardian* and *The Times* in order to study the use of metaphors in more detail and more systematically. As we shall see, the press did not engage as much in creative metaphors as some of the examples discussed above. Interestingly, the newspaper articles we studied stayed much closer to the core scientific metaphors that structure the field of epigenetics, albeit in an ambiguous way.

## Epigenetic metaphors in *The Guardian* and *The Times* (2008–2013)

We used the Nexis^®^ academic database to compile a trend graph in news coverage of epigenetics for the last five years in UK National newspapers, which includes all national broadsheets and tabloids.

**Figure 3.**
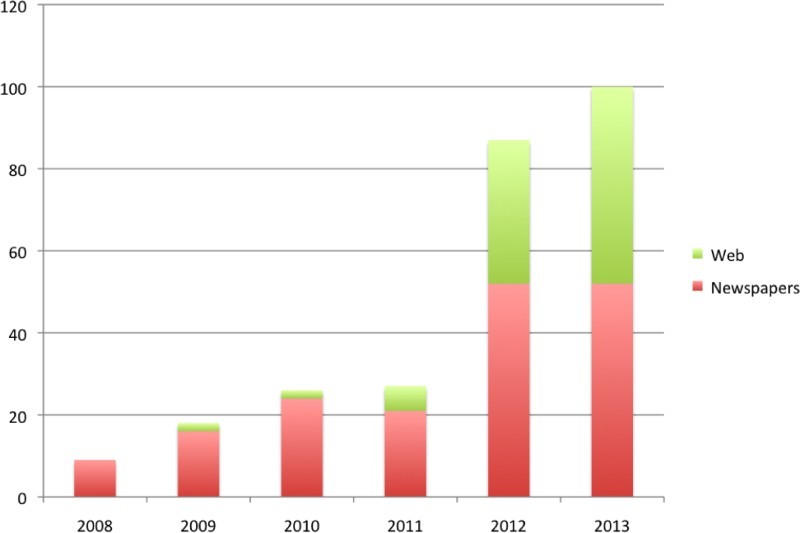
“Epigenetics” in UK National News (Nexis^®^).

As one can see from Figure 3, coverage increased substantially after 2011, driven partly by an increase in web-coverage. We then homed in on a small sub-section of this coverage to make a thorough qualitative analysis possible. We focused on two leading newspapers from two ends of the political spectrum, *The Times* (right-leaning) and *The Guardian* (left-leaning). Both dealt with epigenetics only sparingly, with *The Times* publishing 27 articles between 2008 and 2013 and *The Guardian* 23. Most of the articles in both papers were triggered by publications of books such as Nessa Carey's *The Epigenetic Revolution* (Carey 2012), Tim Spector's *Identically Different* (Spector 2012) and David Shenk's *The Genius in all of us* (Shenk 2010), influential reports such as the one by Harvard's *National Scientific Council on the Developing Child* (2010) or radio programs such as Radio 4's *The first 1000 days: A Legacy for Life*, presented by Dr Porter (2011). There are only very few in-depth articles by science writers and scientists (e.g. Mark Henderson and Hannah Devlin, former and current science correspondents for *The Times*; Alok Jha, science correspondent for *The Guardian*; Robin Lovell-Badge, scientist, Head of Stem Cell Biology and Developmental Genetics at the National Institute for Medical Research; Peter Forbes, a science writer, writing for *The Guardian*’s review pages; there are two pieces by a philosopher, Mary Midgley and a neuroscientist Steven Rose, who unfortunately do not say much about epigenetics).

Topics covered in both papers relate to health (cancer, stress, suicide, autism and nutrition), parenting, social issues (abuse, poverty) and genetics (evolution, selfish gene and nature vs. nurture). Key scientists quoted in the papers include Tim Spector (King's College, London), Robert Plomin (King's College, London), Denis Noble (University of Oxford), Moshe Szyf (McGill University) and David Barker (University of Southampton).

## Method

There is no easy method for identifying metaphors, especially in a field where scientific users might regard a word such as “imprinting” as a neutral and non-metaphorical technical term, while journalists and/or metaphor analysts not working in the field may well see it as metaphorical. We make a distinction between conceptual metaphors and metaphorical expressions (although in general parlance both such “type” and “token” metaphors can be referred to as “metaphors”). Conceptual metaphors allow us to map older or more familiar domains of knowledge, so-called source domains (say electrical circuits, switches), onto newer less familiar domains of knowledge, so-called target domains (say epigenetic phenomena) (Lakoff and Johnson 1980). They can also be seen as conceptual labels for clusters of linguistic expressions. So, the conceptual metaphor *Argument is War* would find expression in or would allow creative expressions like “she shot down his argument,” “he surrendered to her argument,” “she attacked my idea,” “she defended her position” and so on. As one can see, such metaphors can be quite invisible and difficult to detect. This also means that one can easily overlook the work they do, namely shaping the way we think, talk and act. They can illuminate and obscure, open up new vistas or blinker us to alternative ones (see Pauwels 2013; Ginsberg et al. 2014). Metaphors also have political power, in terms of helping to construct a scientific field and in terms of creating public understanding or indeed misunderstanding of that emerging field (Larson 2009).

In order to identify metaphors (i.e. metaphorical expressions in the first instance and overarching conceptual metaphors subsequently), we read all the articles in the two corpora and extracted keywords and candidate metaphors, referring to whole linguistic expressions that are metaphorically used. When deciding whether a word/expression had been used metaphorically, we considered whether it has a more basic, concrete meaning in other contexts (Pragglejaz Group 2007). We systematically lifted the sections containing a metaphor keyword or candidate metaphor from each article and entered them into a spreadsheet, the rows of which represented the articles and the columns recorded the instances of different metaphor keywords and candidate metaphors. We initially intended to record frequencies as well, but the numbers are too small to be meaningful. Metaphorical expressions were compared between the researchers, and ordered into groups and patterns until consensus was reached.

In the following, we shall first study metaphorical expressions in their contexts. We shall then proceed to discuss how clusters of these metaphorical expressions may reveal conceptual metaphors.

## Analysis

### Defining epigenetics

Most articles provided definitions of epigenetics which varied quite widely: “epigenetics, where chemicals in the environment can switch genes in the body on and off” (*The Guardian*, 20 February 2008); “epigenetics, which primarily studies the epigenome, the **protective package** of proteins around which genetic material – strands of DNA – is wrapped” (*The Guardian*, 19 March 2010); “epigenetics is what happens when **genes are actually in action** … in short, epigenetics is where nature meets nurture” (*The Guardian*, 20 August 2011); “Epigenetics, which means “on top of genetics” in Greek, is the phenomenon by which the **genome can remember** these environmental effects” (*The Times*, 7 May 2008); “epigenetics, a phenomenon by which genes are **switched on and off**, and social issues such as parenting, education and the effects of poverty [play a role]” (*The Times*, 31 March 2009) and “‘epigenetics,’ which is used to describe **the inheritance** of changes caused by environmental factors” (*The Times*, 17 September 2008). As one can see, these definitions already contain some metaphors which we will discuss in the following.

Not only do definitions of epigenetics vary widely, but they also convey how difficult these authors find the task of pinpointing what epigenetics is. For example, it is not always clear from media coverage whether epigenetics is a scientific discipline on its own. While some claim that epigenetics is a “phenomenon” (*The Times*, 7 May 2008) or a “process” (*The Times*, 11 August 2010), others describe it as a “theory” (*The Times*, 12 January 2013) or a “study of how environment may modify our genes” (*The Times*, 26 September 2009). Those who acknowledge that epigenetics is a scientific discipline sometimes struggle to explain what it studies. While some journalists refer to epigenetics simply as “the youthful field of epigenetics” (*The Guardian,* 19 March 2010) or “a science [which] is in its early stages” (*The Times*, 7 December 2013), others define it as “a controversial theory which suggests that genes can be switched on or off by environmental factors,” or, more metaphorically, a “**fraught territory** of inherited characteristics” (*The Times*, 12 January 2013).

Epigenetics is not only metaphorically framed as controversial, but it is also seen as strange and even incomprehensible. In one newspaper article, the authors claim that an “epigenetic process [is] a strange mode of inheritance that is only beginning to be properly understood” (*The Times*, 5 February 2009). In one instance, toward the end of an article, the writer simply admits that “I have forgotten what epigenetics actually is” (*The Guardian*, 7 November 2012).

This elusiveness of meaning of epigenetics may of course be analyzed solely in terms of science communication issues which prompted the journalists to frame epigenetics as controversial, incomprehensible or strange. It might be useful however to put these kind of statements in a wider context. As Goldberg et al. remind us, “[h]istorically the word “epigenetics” was used to describe events that could not be explained by genetic principles. ( … ) Over the years, numerous biological phenomena, some considered bizarre and inexplicable, have been lumped into the category of epigenetics” (Goldberg, Allis, and Bernstein 2007, 635). Thus the struggle with communicating the essence of epigenetics might reflect current problems faced by the scientific community itself. As Bird has noted, “Geneticists study the gene; however, for epigeneticists, there is no obvious ‘epigene’” (Bird 2007, 396). And this lack of the obvious “epigene” makes epigenetics a puzzle. Let us now see how metaphorical expressions try to deal with this puzzle.

### Clusters of metaphorical expressions

Two important clusters of metaphorical expressions emerged which seem to be at the core of epigenetic research. These two metaphors are the switch or switching metaphor and the mark, tag or label metaphor. We discuss later whether they can be called conceptual metaphors. Other metaphors of, for example, memory, inheritance and music cluster around them.

The following examples illustrate the use of the switch metaphor in explaining epigenetics: “epigenetic, where chemicals in the environment can **switch** genes in the body **on** and **off**” (*The Guardian*, 19 February 2008); “‘epigenetics’ – the mechanism by which genes can be **switched on** and **off**” (*The Times*, 13 June 2012) and “genes are **switched on and off** by complex chemical modifications, a process known as epigenetics” (*The Times*, 11 August 2010).

The switch metaphor is not new; it has quite a venerable history. It was used very early on in the history of epigenetics, for example by Waddington in the 1940s. Julian Huxley wrote a review of Conrad H. Waddington's *Principles of Embryology* (Waddington 1956) in 1956 for the journal *Nature* (and as far as we can ascertain, this was the first time that the term “epigenetics” was used in that journal):

Furthermore the metaphor of unstable equilibrium is unsatisfactory: bicompetence is best assimilated to those numerous epigenetic phenomena where a switch-mechanism is operative. In such cases, alternative causes or stimuli switch development into alternative pathways, each of which has been sharply limited or homoeostasized (stabilized) by past selection. As with a **railway switch**, there need be no instability, whether on the single **pathway** before reaching the **switch**, or on either of the alternative **tracks** along which the process may continue. (Huxley 1956, 807, highlighting added)

Interestingly, Huxley uses the switch metaphor in the context of Waddington's eminent metaphor of the epigenetic landscape, a central metaphor of early epigenetics, which is however almost totally absent from current popular epigenetic discourse and seen as not particularly useful by scientists communicating about epigenetics (see Bird 2013b). More interestingly still, Huxley's switch metaphor uses as a source domain our cultural experience of railways, whereas current uses of the switch metaphor tap into our experience of light switches, which enable us to switch light on and off. When writing this article at the beginning of 2014, we also found a reference to a “dimmer switch” and a “thermostat”: Hannah Devlin reported for *The Times* on a new study carried out by Spector's group under the title “**Dimmer switch** may lead to better pain killer” (*The Times*, 5 February 2014). Progress in technology leads to changes in metaphorical framing. The second core metaphor is that of the epigenetic “mark” or “tag” left on our DNA. These marks are sometimes called “post-it notes” or “bookmarks” outside our small corpus, as well as, less metaphorically, “epimarks.” These marks determine which parts of our DNA get switched on or off. They are left on our DNA by the life we lead, that is, whether we smoke, eat too much or too little food, are exposed to stress and abuse and so on. These epimarks could also get passed on to children. In our corpus, we found a few uses of this “mark” or “marker” metaphor, as for example here: “Professor Szyf said: ‘It's possible the changes in epigenetic **markers** were caused by the exposure to childhood abuse’” (*The Times*, 7 May 2008). And, focusing directly on environmental influences like smoking, “‘Epigenetic **markers** may be the mechanism behind how nicotine-induced stems are transmitted from one generation to the next,’ said lead author, Dr Virender Rhan, from UCLA” (*The Times*, 30 October 2012).

These marks or tags are, as we have seen, linked to the switch metaphor, but they are also linked to another metaphor, namely the metaphor of memory. As we all know, post-it notes are there for us to remember things and also to remember which page of a book to read. In the same way, epigenetic marks or tags ensure that our DNA remembers or, indeed, forgets certain environmental impacts. This memory may, as some claim, last across several generations. Here are some examples of this type of memory metaphor found in our corpus: “Epigenetics, which means ‘on top of genetics’ in Greek, is the phenomenon by which the genome can **remember** these environmental effects” (*The Times*, 7 May 2008); “‘It is remarkable that maternal diet can mark our genes so they **remember** events in early life,’ said Miguel Constancia, a co-author of the paper” (*The Guardian*, 8 March 2010).

This idea of a transgenerational genetic memory can lead to new speculations about blame and responsibility (Nerlich 2012). In the past, some people tried, for example, to blame their obesity on the bad genes they had inherited. With epigenetics, the blame shifts from the bad genes, for which our ancestors cannot directly be blamed, to a bad epigenome, for which they can be blamed, as it may be the outcome of a bad lifestyle or traumatic life. As one commentator of Jewish extraction said: “I am loving epigenetics, the idea, loosely put, that your **genes have** “**memories**” … The good thing about epigenetics is that it allows me plausibly to **blame** being overweight on the Nazis” (*The Times*, 7 December 2013).

The metaphor of “memories” of past experiences being embodied at molecular level also helps to convey the frequently repeated claim that epigenetics is about genetic “inheritance” or genealogy. Interestingly, two recent popular books on this subject (and one of them is reviewed in our media texts sample) have the word “inheritance” in their titles (i.e. Nessa Carey's book *The Epigenetics Revolution: How Modern Biology is Rewriting Our Understanding of Genetics, Disease and Inheritance* and Richard Francis's book *Epigenetics: The Ultimate Mystery of Inheritance*). We found similar claims in our corpus. For example, as one article claimed, “Here it is worth introducing a word with which we may all grow familiar over the next two or three years – ‘epigenetic’, which is used to describe the **inheritance** of changes caused by environmental factors” (*The Times*, 17 September 2008). According to another piece, “epigenetics is beginning to reveal how highly stressed parents can **bequeath** a propensity for anxiety and depression through the genes that they **pass to** their children” (*The Times*, 4 September 2010). One journalist even claimed that “some epigenetic changes are so long-lasting they cover several generations: they can be **inherited**. This flouts one of biology's most cherished dogmas – taught to all students – namely that changes **acquired** during life cannot be **passed on** – the heresy of Lamarckism” (*The Guardian*, 20 August 2010).

This particular text prompted a response from the evolutionary biologists Deborah and Brian Charlesworth. They pointed out that “[i]f epigenetically caused differences are transmitted from parents to offspring, their effects are thus tiny and cannot account for much of what happens in evolution.” It is noteworthy that in this context epigenetics is framed as a kind of new vehicle for inheritance. In this sense, texts on epigenetics repeat and reframe the same preoccupation that constituted a constant theme in texts on genetics, namely the question of inheritance and the future of subsequent generations (see Müller-Wille and Rheinberger 2012).

However, there was also talk of epigenetic “marks” being “**erased**,” “**silenced**,” “**masked**” or “**unmasked**.” Here the notion comes to the fore that we can shape our genetic destiny through shaping the environment in which we live or the lives we live in any environment which leads to us being able to shape and change our epigenome.

This theme of genetic or epigenetic plasticity was especially explored through the use of a variety of musical metaphors, such as: “epigenetics – the ‘**notation**’ that determines how the **notes** for the DNA sequence are **played** during plant and animal development” (*The Times*, 5 August 2009);

If the genetic code were a **keyboard**, the epigenome would be the **pianist**. Different **chords** become the various cell types, and all the notes have to be **played perfectly to produce a** healthy **human being**. Damage to the epigenome – the pattern of chemicals that controls our genes – has been linked to medical conditions as diverse as asthma, schizophrenia and cancer. (*The Guardian*, 15 October 2009)

Overall, there was a rather paradoxical framing of genes as active and passive at the same time, as being controlled through epigenetics (“epigenetic instructions,” *The Times*, 26 August 2011) and as issuing instructions themselves: “genes have **to be activated** at the right time and place, and this is **controlled** in part by processes often referred to as ‘epigenetics’” (*The Times*, 6 August 2008);

Epigenetics is what happens when **genes are actually in action ** … **responding** to hormones and environmental stress … . In this process genes are modified slightly and **act** differently from that point on … The **cell tells the DNA what to do** just as much as the **DNA instructs the cell ** … Genes don't just **issue instructions**: they **respond to messages** coming from other genes, from hormones and from nutritional cues. (*The Guardian*, 20 August 2011)

Most interestingly, there is some talk of changing this process of “instruction” and thereby avoiding disease: “In other words, a dodgy gene, caught early, **can be taught** to **resist** rather than **adopt** the disease for which it is heading” (*The Times*, 17 September 2008). Here we find again an indication of hope that genes, through epigenetics, can be made flexible and can be changed in an advantageous way.

This means that the old metaphor of the “blueprint” is still useful but is coming to be seen as more flexible and dynamic than before.

They [identical twins] share the same DNA and genes that make up the genome – the **blueprint of life ** … The same blueprint can be **read** in different ways: genes have to be **activated** at the right time and place, and this is **controlled** in part by processes often referred to as “epigenetics” (*The Times*, 6 August 2008)

“ … far from being a **static blueprint**, our DNA is open to continual influence by external factors” (*The Times*, 20 March 2010).

However, hopes are invested in epigenetics which go beyond a new type of control over disease. Some hope that epigenetics may provide policy-makers with a new way of getting to grips with, indeed getting control over, social problems, such as poverty, abuse and violence.

This was particularly apparent in an interview carried out for *The Times* by Magnus Linklater, the Times’ Scottish correspondent, with Sir Barry Burns, Scotland's Chief Medical Officer (*The Times*, 12 January 2013). Epigenetics seems to provide a biological lever that enables policy-makers to gain control over physical, mental and social health and to reduce health inequalities. This discourse is based partly on the switch metaphor, as used by Sir Barry here: “‘This is very deep and uncertain territory,’ he admits. ‘Your genes are your genes, but we now realize that you can have all the **good** genes that you like, but if the **right** ones are **switched off** and the **wrong** ones are **switched on**, you have a very different future.’” Epigenetics is here seen as a way to create “better” futures, to avoid “bad” futures by turning off the mechanism, epigenetics, which may be involved in transmitting bad influences coming from the environment to future generations.

Epigenetics is almost seen as a socio-political control switch: “If you want to turn it off [the bad influence of epigenetics across generations], we have **to turn it off** by tackling the causal influences, which is family disintegration and poor parenting” (Sir Barry Burns, quoted in *The Times*, 12 January 2013). This political and medical control can be achieved through regulation, in the sense of social policy interventions. Indeed, Sir Barry advocates a “place for regulation where things are out of control.” Indirectly perhaps there is a hope expressed here that regulating unruly genes through manipulating social environments may help regulate unruly or out of control parts of society.

This hope of a new type of environmentally and biologically mediated political control seems to be nurtured by some scientists such as Dr Oliver Davis, in this instance, who claims that “[y]our **genes are not your destiny ** … It is all about what you can do environmentally to mitigate or enhance the effect of genes” [Dr Oliver Davis, quoted in *The Times*, 13 June 2012). This seems to imply that not only governments but individuals can also change their destiny by changing their environments, controlling their genes and thus their lives.

Part of this “environment,” and for some almost the most important part, is the rather primordial environment of the womb, which is the focus of one field that is linked to epigenetics, namely the fetal programming hypothesis promoted by Professor David Barker. Here we find talk of “securing one's offspring's future – possibly even before conception” (*The Times*, 26 August 2011). More importantly, there is talk of controlling the “**build quality**” of the fetus within the womb, metaphorically framed as a “factory”: from “ … poor nutrition and stress to smoking, drugs and alcohol … Once a **build phase** is complete, and **quality compromised**, it cannot be improved after the child leaves the ‘**factory**’” (*The Times*, 26 August 2011). And another article in late 2013 by Dr Mark Porter (a medical doctor and journalist) is entitled: “Your child's future health is determined in the womb” (*The Times*, 15 October 2013). Surprisingly, the articles we looked at did not use a rather popular metaphor created by Barker himself, namely of the “thrifty phenotype” (Hales and Barker 1992), which drew on Neel's (1962) idea of a “thrifty genotype.” This metaphor would deserve further research as it can have direct impact on parenthood, nutritional habits of the mother and put extended genealogical pressure on parents.

Overall, the discussion of epigenetics in the two papers is quite measured and one can only occasionally find some hype and hyperbole, as for example in an article by Peter Forbes for *The Guardian* which says in its title: “Epigenetics is one of the **keys** to explaining the mystery of life” and claims in the article that epigenetics “will revolutionise science” (*The Guardian*, 11 August 2012).

### Conceptual metaphors and a puzzle

When scrutinizing the metaphorical expressions we found more closely, one can begin to put them into various overarching groups or “conceptual metaphors” (Lakoff and Johnson 1980). One can also see how mappings between source domains and target domains are performed that make these metaphors work – or not. In the previous section, we have focused on “metaphorical expressions,” such as “switching,” “marking,” “tagging,” “inheriting,” “remembering” and so on. We shall now try to ascertain how they cluster together around a smaller number of overarching conceptual metaphors.

Three major conceptual metaphors seem to structure the media discourse about epigenetics that we have observed. But unlike in the context of genetics and genomics, where one can observe relatively simple and transparent mappings between source domains and target domains that lead to a feeling of an increase in understanding of what genes and genomes are, the epigenetics mappings highlight a problem with the target domain. Of course, target domains are always underspecified in comparison to source domains; otherwise a metaphorical mapping would not be needed, as the metaphor fills an apparent knowledge gap. However, compared to “the genome is the book of life,” which allows readers to imagine leafing through the genome, extracting information that provides deep insights into being human, the epigenetic metaphors seem to not be so seemingly transparent and enlightening, as we shall see. This may be indicative of a scientific field still being in a state of flux and under construction.

In the case of genomics, the target domain is “the gene” or “the genome” and the source domains are familiar everyday objects, such as books, which make the unfamiliar, namely the gene or genome, seem familiar and comprehensible. In the case of epigenetics, we have various source domains (such as machines, mechanical devices and human agents) but we have no very clear target domain.

The three overarching conceptual metaphors based on source–target mapping are the following:

X is a human agent (acts, remembers, controls, instructs, responds, teaches, bequeaths, etc.).

X is a mechanical agent (switches, marks and tags).

X is a human agent using a mechanical agent (piano player – piano).^2^

The question is: What is X? X may be the epigenome. However, the mapping process fails, as what is familiar is mapped not onto something relatively unfamiliar, like the gene, but onto something altogether vague and ill-understood. X seems to mark the spot where epigenetic science is still struggling and also the spot where making epigenetics public is still proving difficult.

The vagueness of the concept of epigenetics and the difficulty of mapping it clearly onto something familiar may stem from several reasons. As Robin Holliday, a distinguished epigeneticist, has once noted, Waddington coined the term epigenetics to link the separate disciplines of genetics and developmental biology (Holliday 2006, 76). In Waddington's words, “We certainly need to remember that between genotype and phenotype, and connecting them to each other, there lies a whole complex of developmental processes” (Waddington 2012[1942], 10). And even today, in spite of the many debates about its possible meanings and scope, epigenetics is interpreted as a “bridge between genotype and phenotype” (Goldberg, Allis, and Bernstein 2007, 635), while epigenetic mechanisms are viewed as “mediators between environment and the genome” (Stöger 2008, 159).

We suggest that this particular state of being in-between is reflected in the fact that there are no “grand” or generally accepted metaphors yet for epigenetics. Some may therefore think of this state of affairs as a “muddle” (Keller 2010), “muddle” that is compounded by the fact that epigenetics and debates surrounding it are often framed in terms of an old dichotomy of nature versus nurture, where epigenetics is presented as a solution to this eternal philosophical dilemma. But what exactly that could mean and entail is not at all clear.

Some articles in our corpus reflected this struggle to find a settled meaning for epigenetics. In one article, the author tried to convey the intermediary feature of epigenetics, that is, “epigenetic processes **mediate** effects of social adversity that persist into adulthood and are known to enhance suicide risk” (*The Times*, 7 May 2008). Other texts use the well-known schemata of nature versus nurture debate, where nature stands for genes and nurture for environment, to try to explain what epigenetics is. As we have seen, some tend to describe epigenetics in relation to genes, as “changes to the genes” or “genes in action” (*The Guardian*, 20 August 2011). Alternatively, they framed epigenetics as akin to some kind of ill-defined environment. In one instance, however, epigenetics is defined in relation to both genes and environment/experience:

**genes in action** do some strange things that we are only just beginning to understand – identical genes can diverge in their expression during the course of a lifetime. This is epigenetics. It is now generally accepted that **personal experience can change our genes**. (*The Guardian*, 11 August 2012)

Some authors almost equate epigenetics with food, or effects of a particular diet which is illustrated in the following example: “epigenetics (**the effect of what your grandfather did or didn't eat for breakfast)** has been shown to be transmitted down the generations” (*The Guardian*, 9 June 2013).

Many texts in our sample tend to define epigenetics in a close relation to environment, to the point that epigenetics starts to be metaphorically framed *as* environment. While some texts employ quasi-neutral terms of “environmental factors” (*The Times*, 12 January 2013) or “environmental effects” (*The Times*, 7 May 2008), others use less neutral expressions, for example, “epigenetics – the analysis of environmental effects on genes – that shows how the **lifestyle choices** of grandparents and even great-grandparents can have genetic consequences down the generations” (*The Times*, 30 October 2012). Or “any high school student knows that genes are **passed on** unchanged from parent to child, and to the next generation and the next. **Lifestyle** cannot change heredity. Except now it turns out that it can … ” (*The Guardian*, 19 March 2010). This type of framing is not unusual – many commercial websites represent epigenetics as a new lifestyle and health enhancement option. Our analysis shows that through such framing the concept of epigenetics is made extremely malleable also in the media coverage.

## Discussion and conclusions

At the beginning of this paper, we briefly reviewed grand metaphors and grand claims made during the heydays of genomics. Let us now come back to these metaphors and claims and review them in the light of our small qualitative study of metaphors used to convey the importance of epigenetics in two leading UK newspapers.

Instead of grand genomic metaphors of the “book of life” type, we found numerous smaller metaphors being used to convey core messages about epigenetics. Especially popular are the switch and the mark/tag metaphors, accompanied by words such as “process” and “mechanism,” as well as “control.” Not only do they highlight the dynamic nature of epigenetics over the more static perspective of the genome as a book, map or blueprint, but also foreground hopes of control over and regulation of human physical and social health.

While in the past, genes were framed by some scientists as our destiny and having power over us, we now see a discourse emerging where genes are no longer our destiny and where we seem to have power over our genes, a power mediated by the environment we live in and shape through our actions. The specter of genetic determinism seems to have been banished. This entails different notions of blame and responsibility. On the one hand, we can blame our ancestors and/or the environments in which they lived for our current state of health. On the other hand, we now have responsibility not only for our own health, but also for our fetuses’ health and, possibly, for the health of future generations.

Whereas in classical genomics, promises were made about personalized medicine, the promissory discourse has now shifted to populations and public and social health and the hope is that (early) interventions into social care, housing, parenting and pregnancy may alleviate all sorts of personal and social ills.

Whereas in the past the direction of claims making went from the inside out, claims making now goes from the outside in. Instead of making people better and healthier by changing their genetic make-up, the focus is now on making people better and healthier by changing the environments they grow up and live in. There is also much more of a focus on time and space, in terms of epigenetic effects potentially spanning several generations in time and spanning everything from the inside of the womb to social and cultural environments in space.

Most importantly, we found that while grand metaphors in genetics and genomics were based on relatively transparent mappings between source domains (books, computers, programs and maps) and target domains (genes and genomes), such seemingly transparent mappings were not obvious in epigenetics. There are various source domains that are being exploited, such as machines or mechanical devices on the one hand and human agents on the other, indicating that the target domain is still being constructed and open to interpretation. This situation poses a challenge when conveying the importance of epigenetics to various publics, including policy-makers who might have been more receptive to hearing about a “wondrous map” and “the book of life” (Nerlich, Dingwall, and Clarke 2002). However, there is enough metaphorical “stuff” there for lay people and policy-makers as well as social scientists to be able to imagine better futures or, indeed, for thinking about the social, ethical, political and personal implications of epigenetics. In this respect, a “plurality of (small) metaphors is perhaps not a bad thing” (Larson 2009).

In the case of genes and genetics, “historically, it has been difficult to pin down exactly what a gene is or does” (Turney 2005, 808). Even today as both natural and social scientists point out (see Turney 2005; Noble 2006), genes can mean different things to different scientists. But despite the slipperiness of the gene concept, there are grand metaphors associated with genes, such as “the book of life” which emerged as a consequence of various developments in genetics, in particular following the so-called Central Dogma, according to which DNA makes RNA and RNA makes protein (see Leavitt 2010).

With epigenetics, the situation is different: like with genes, epigenetics might mean different things to different scientists, but as yet there are no grand epigenetic metaphors. Following Larson (2009), it might be argued that numerous smaller metaphors open up the scope for various interpretations and allow a more nuanced approach to the phenomena that epigenetics study. It might also indicate that epigenetics is still waiting for its scientific “breakthroughs” and great debates which might “solidify” some of the epigenetic metaphors. As Lenny Moss has argued, “the idiom of the language-of-the-gene became written not by those whose hypotheses were successful but rather by those whose metaphors were successful” (Moss 2003, xvii). It remains to be seen which metaphor – if any – will attain the grand metaphor status, one that can unite the field and span scientific and popular discourse.

In the meantime, the landscape of epigenetics, framed by old and new metaphors, provides a new space for the emergence of societal, psychological, political hopes and fears and new ways of apportioning blame and responsibility. There is hope that we can now not only read and write the book of life, but also write a script or musical score according to which the lives of future generations can play out, hopefully in better ways, in terms of both physical and social health. But who writes the score and who directs the music: individuals, communities, politicians? There is fear that we are entering a new age of eugenics or “molecular determinism” (Pickersgill and Fletcher 2012). Ultimately, we, but in particular mothers and fathers, may have to shoulder new responsibilities, because what we do now (whether willingly in terms of diet or smoking, or unwillingly in terms of living in harsh or violent social conditions) may impact not only on the lives of our children but also our children's children. We also have to find new ways of thinking about blame. Blaming our parents for giving us the wrong genes is no longer the only way to deflect blame for our behavior or our state of health; we can now also blame our parents’ parents (and the politicians who ruled/ruined their and our lives), starting a whole intergenerational blame-game. This is of course only the case if transgenerational epigenetic effects can be proven to exist. The problem is we do not quite know yet. As a specialist in epigenetics, Professor Edith Heard said in an interview with *The Observer*:

Even our epigenetic changes are genetically driven. The code of genetics is the code. It's the only code.” But now with epigenetics, “people are hoping we can pray our way out of faulty genes” … “It's our duty as scientists to pass on the right messages. I don't want to say epigenetics isn't exciting … [but] there's a gap between the fact and the fantasy. Now the facts are having to catch up. (*The Observer*, 23 June 2013)

Similarly, Tolwinski (2013) found a great variety of opinions about epigenetics amongst epigenetic scientists, a variety that is not quite mirrored in the social science studies dealing with epigenetics. She argues:

 … that the varied discourse about epigenetics means that its trajectory will be far more complex and contested than some social scientists suggest. Even if the most optimistic perspective that understands epigenetics as ‘Biology 2.0’ does persist, the end result may not be one that pleases anti-reductionist and anti-determinist sentiments. (Tolwinski 2013, 17)

Our analysis of metaphors has shown that epigenetics is still a field in flux and a very complex one at that. It can mean different things to different people and it has a variety of definitions, as we have shown in our media analysis. An array of metaphors, exploiting a number of familiar source domains, are emerging to talk about epigenetics, but they have no common “target,” making it difficult to communicate about this new field and its social, ethical and political implications. This plasticity of the field itself has to be acknowledged before building up promises and hopes around epigenetic plasticity.

## Disclosure statement

No potential conflict of interest was reported by the authors.

## Notes

1. We highlight metaphorical expressions in bold.

2. In metaphor research, conceptual metaphors such as *Life is a journey or arguments are war* are marked through the use of small capitals. This typographical convention allows linguists to distinguish between metaphorical expressions found in real language use (which we highlighted in bold) and conceptual metaphors that underlie these expressions but are not found as such in the texts studied.
